# Pharmacological Evaluation of the SCID T Cell Transfer Model of Colitis: As a Model of Crohn's Disease

**DOI:** 10.1155/2012/412178

**Published:** 2012-02-19

**Authors:** Thomas Lindebo Holm, Steen Seier Poulsen, Helle Markholst, Stine Reedtz-Runge

**Affiliations:** ^1^Department of Immunopharmacology, Novo Nordisk A/S, 2760 Måløv, Denmark; ^2^Department of Medical Anatomy, The Panum Institute, University of Copenhagen, 2200 Copenhagen, Denmark; ^3^Department of Haemophilia Biology, Novo Nordisk A/S, 2760 Måløv, Denmark

## Abstract

Animal models are important tools in the development of new drug candidates against the inflammatory bowel diseases (IBDs) Crohn's disease and ulcerative colitis. In order to increase the translational value of these models, it is important to increase knowledge relating to standard drugs. Using the SCID adoptive transfer colitis model, we have evaluated the effect of currently used IBD drugs and IBD drug candidates, that is, anti-TNF-*α*, TNFR-Fc, anti-IL-12p40, anti-IL-6, CTLA4-Ig, anti-*α*4*β*7 integrin, enrofloxacin/metronidazole, and cyclosporine. We found that anti-TNF-*α*, antibiotics, anti-IL-12p40, anti-*α*4*β*7 integrin, CTLA4-Ig, and anti-IL-6 effectively prevented onset of colitis, whereas TNFR-Fc and cyclosporine did not. In intervention studies, antibiotics, anti-IL-12p40, and CTLA4-Ig induced remission, whereas the other compounds did not. The data suggest that the adoptive transfer model and the inflammatory bowel diseases have some main inflammatory pathways in common. The finding that some well-established IBD therapeutics do not have any effect in the model highlights important differences between the experimental model and the human disease.

## 1. Introduction

The two inflammatory bowel diseases (IBDs) ulcerative colitis (UC) and Crohn's disease (CD) affect more than 3.6 million people in the Western world, resulting in a marked decrease in the patients' quality of life [1, 2]. The aetiology is poorly understood, but it has become clear that genetic, microbial, and environmental factors all play a role [3]. A massive effort is taking place to develop new and better therapeutics, and the development of tumor necrosis factor-*α* (TNF-*α*) antagonists has ameliorated the disease in a large proportion of especially CD patients [4]. However, about one third of the CD patients do not respond to anti-TNF-*α* treatment and among the primary responders, about one third loose response or become intolerant to the treatment [5], thus leaving many IBD patients with inadequate therapeutic options.

New IBD drugs and drug candidates include anti-interleukin (IL)-12/-23 (e.g., ustekinumab, briakinumab), cytotoxic T-lymphocyte antigen 4 immunoglobulin (CTLA4-Ig, abatacept), anti-IL-6R (tocilizumab), anti-interferon *γ* ((IFN-*γ*), fontolizumab), anti-*α*4*β*7 (vedolizumab), anti-*α*4 integrin (natalizumab), anti-IL-2-R*α* (daclizumab, basiliximab), antigranulocyte macrophage colony-stimulating factor (anti-GM-CSF, sagramostim), anti-intercellular adhesion molecule 1 (anti-ICAM-1, alicaforsen), rIL-18 binding protein (tadekinig-*α*), IP-10/CXCL10 (MDX-1100), anti-CD3 (visilizumab), and anti-CD40L (TNX 100) [6, 7]. These compounds aim at targeting specific immunological mechanisms like cellular adhesion (anti-*α*4*β*7, anti-ICAM-1) and costimulation (CTLA4-Ig, anti-CD40L), key cytokines (anti-IL-12/-23, anti-IL-6R, anti-IFN-*γ*) or cells (anti-IL-2R*α*/CD25, anti-CD3), or have specific immuno-stimulatory (GM-CSF) or -inhibitory (rIL-10) effects.

Animal models are essential for dissecting the role of the pathological mechanisms in IBD as well as for assessing the therapeutic effect of intervening with these pathways [8]. To what extent the data from animal models can be translated into the clinic differs among the various models depending, for example, on the model's etiopathogenesis and main drivers of disease. There is no single model which adequately mimics either UC or CD, and to be able to translate findings from a model to the human disease, it is important to know the model's central pathological mechanisms and immunological pathways.

Adoptive transfer of a subset of CD4^+^ T cells to syngeneic SCID or Rag-knock-out mice, results in the development of a chronic, progressive colitis and wasting disease as first described by Morrissey et al. and Powrie et al. [9, 10]. The colitis symptoms share several features with both CD and UC (e.g., chronic, progressive disease with diarrhoea and weight loss, heavily inflamed colon—occasionally transmural damage, loss of mucus from goblet cells, Th1/Th17 dominated cytokine profile as found in CD (IFN-*γ*, TNF-*α*, and IL-23). The model has been extensively used for studying the immunologic background for the disease as well as testing new IBD drug candidates [11–14]. We have previously described in detail the development of colitis following adoptive transfer of CD4^+^CD25^−^ T cells [15]. Briefly, in our hands, the adoptively transferred cells expand rapidly and the mice begin to develop colitis within the first two weeks. At week three, the disease is normally fully developed with weight loss, loose stools, increased white blood cell (WBC) count, and a both thickened and shortened colon. The disease progresses rapidly and by week 5 most mice have developed severe colitis requiring a termination of the study. This synchronized and predictable development of colitis makes it possible to conduct both prevention and intervention studies.

The aim of this study was to analyze the model with respect to its usefulness in efficacy studies of new IBD drug candidates by evaluating the effect of known and potential IBD therapeutics in the model. We have decided to study a number of compounds, which each has a specific inhibitory effect on a central proinflammatory pathway. In addition, we have included some established IBD therapies, suggested to ameliorate IBD by a broad spectrum of mechanisms.

## 2. Materials and Methods

### 2.1. Materials

Human CTLA4-Ig (abatacept, Orencia, Bristol-Myers Squibb), human tumor necrosis factor receptor Fc (TNFR-Fc) (etanercept, Enbrel, Wyeth), enrofloxacin (Baytril, Bayer, equivalent to ciprofloxacin), metronidazole (Flagyl, Sanofi-Aventis), cyclosporine (Sandimmun, Novartis). All surrogate antibodies, that is, anti-TNF-*α* (clone XT3.11, rat IgG1), anti-IL-12p40 (clone C17.8, rat IgG2a), anti-IL-6 (MP5-20F3, rat IgG1), anti-*α*4*β*7 (clone DATK32, rat IgG2a), and isotype controls (cIg) (rat IgG2a clone 2A, rat IgG1 clone HRPN and human IgG1-Fc) were from BioXCell, West Lebanon, New Hampshire, USA.

Dynabeads, Mouse CD4 (L3T4), and DETACHaBEAD Mouse were from Dynal, Oslo, Norway, and CD25 MicroBead kit from Miltenyi Biotech, Bergisch Gladbach, Germany. The antibodies used for FACS analysis were PerCP-conjugated anti-CD4 (L3T4) from BD Pharmingen and FITC-conjugated anti-CD45.2 (104) from eBiosciences, CA, USA.

### 2.2. Mice

C.B-Igh-1b/IcrTac-Prkdcscid (C.B-17 SCID) and BALB/cAnNTac female mice (8–10 weeks) bred under SPF conditions (M&B Taconic, Denmark) were housed at Novo Nordisk A/S. Pathology screening was conducted according to FELASA guidelines. The animal studies were approved by the Danish Animal Experimentation Inspectorate.

### 2.3. Induction of Colitis

For induction of colitis, CD4^+^CD25^−^ T cells were adoptively transferred from MHC-compatible Balb/c mice to C.B-17 SCID recipients as described previously in [15]. In brief, Balb/c splenocytes were positively selected for CD4^+^ T cells using Dynabeads and DETACHaBEAD and depleted of CD4^+^CD25^+^ cells using the CD25 MicroBead kit. The purity of the cells was always analyzed by flow cytometry before reconstitution (>98% of the CD4^+^ cells were CD25^−^). The recipients were reconstituted with 300,000 cells by i.p. injection. Peripheral blood from all mice was subjected to flow cytometric analysis 2 or 3 weeks after transfer, and only mice with CD4^+^ T cells (indicating successful transplantation of cells) were included in the study.

### 2.4. Experimental Setup

**Figure 1:**
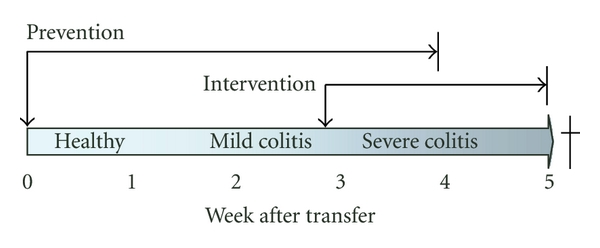
The adoptive transfer colitis model and treatment design. For prevention studies, the mice were treated from the day they were adoptively transferred with CD4^+^CD25^−^ T cells and until sacrificed when the disease is fully developed (three or four weeks after transfer). For the intervention studies, the treatment was initiated at week three after adoptive transfer. The treatment was continued for two weeks until sacrificed at week five.

The drugs tested, as well as the doses and dosing regimens are described in Table 1. For prevention studies, the mice were treated from the day they were adoptively transferred with CD4^+^CD25^−^ T cells and until sacrifice when the disease was fully developed (three or four weeks after transfer, Figure 1). For the intervention studies, the treatment was initiated at week three after adoptive transfer, when the CD4^+^ T cells had expanded and caused colitis in the recipients. The treatment was continued for two weeks until sacrifice at week five. The control groups for the biologics (except for TNFR-Fc) were treated with the relevant control immunoglobulin (cIg), that is, rat IgG1 for anti-TNF-*α* and anti-IL-6, rat IgG2a for anti-IL-12p40 and anti-*α*4*β*7, and human IgG1-Fc for CTLA4-Ig. The vehicle groups for cyclosporine and antibiotics received sterile H_2_O (Table 1). In the study with antibiotics, we also included a group, which was not reconstituted but received treatment, since we suspected that disturbance of the gut microflora in itself could have a marked effect on the measured disease parameters. We used the same doses and dosing frequencies for the various compounds in the prevention and intervention studies (Table 1). The selection of doses and dosing frequencies were based on either literature describing efficacious treatment in various colitis models or based on our experience with efficacious treatment in the collagen induced arthritis model [16–23].

### 2.5. Monitoring of Disease

Body weight was determined three times weekly, and mice were sacrificed if they lost more than 20% of their initial body weight. Fecal consistency was evaluated before the start of treatment and at the termination of the study using a semiquantitative score (normal stool = 0; slightly soft = 1; soft but formed = 2; not formed = 3; liquid stools or no feces in colon at sacrifice = 4) as previously described [15]. The number of WBC per liter was analyzed in samples (20 *μ*L) of EDTA-stabilized peripheral whole blood, using a Medonic CA 620 (Boule Nordic, Denmark) blood analysis apparatus according to the manufacturer's instructions.

### 2.6. Postmortem Analysis

Prior to sacrifice, the mice were anesthetized and blood from the periorbital venous plexus was collected in EDTA-containing tubes. After sacrifice, the colon was excised, rinsed gently with saline, and the weight and length recorded. The colonic weight-to-length ratio (W : L) was previously shown to correlate strongly with the clinical and histological severity of disease [15]. The colon was opened longitudinally, mounted on a plastic plate, and fixed overnight in 4% paraformaldehyde.

### 2.7. Histology

**Figure 2:**
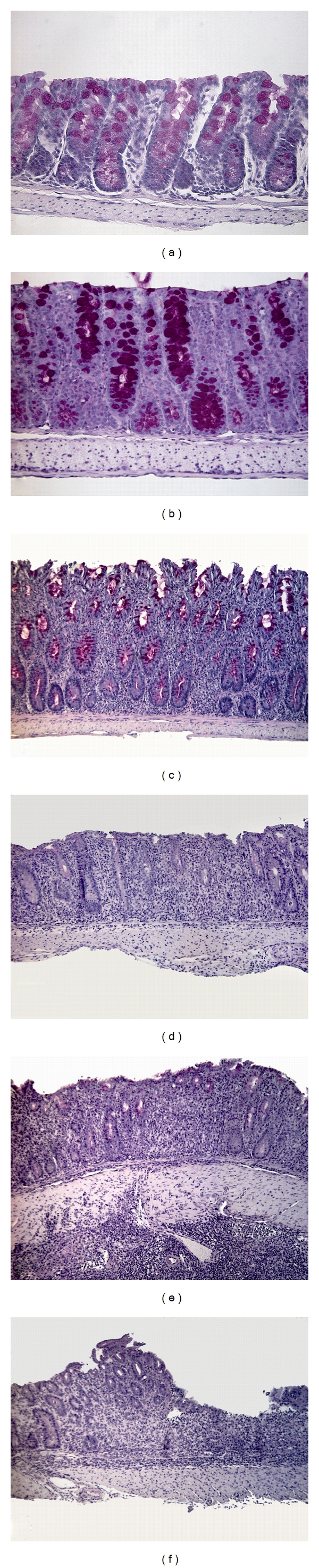
Histological changes in colon after adoptive transfer. Representative photomicrographs of histological changes leading to a progressively higher score from (a) to (f): (a) normal, (b) mild inflammation restricted to the mucosa, (c) moderate mucosal inflammation, (d) severe inflammation extending to submucosa, moderate to severe crypt degeneration, (e) severe transmural inflammation, moderate to severe crypt degeneration, (f) as (e) but with ulceration. Original magnification ×25 for (a-b) and ×10 for (c–f).

Longitudinal segments of tissue representing essentially the entire length of the transverse and distal colon (where the inflammation is mainly located) were embedded in paraffin. A section (7 *μ*m) of the transverse and the distal colon from each animal was stained with hematoxylin and eosin/periodic acid Schiff (H&E/PAS) and analyzed by light microscopy. A total histological score was calculated for each animal as described previously [24] and shown in Figure 2. Briefly, the samples were assigned a score (0–3 or 0–4) according to the severity (none, mild, moderate, severe) and extent (none, mucosal, submucosal, transmural) of inflammation, degree of crypt damage (basal 1/3 damaged, basal 2/3 damaged, crypts lost—epithelium intact, crypts lost—epithelium lost), and percentage of tissue affected (0, 1–25%, 26–50%, 51–75%, 76–100%).

The histological analyses were performed in a blinded fashion with respect to the treatment groups.

### 2.8. Statistical Analysis

Fecal consistency score and histological score are shown as median (range) and analyzed using the Mann-Whitney *U*-test. WBC count, body weight at postmortem, and colonic weight : length ratio are shown as mean ± standard error of the mean (SEM) and analyzed by Student's *t*-test using Welch's correction for unequal variances. Differences were considered statistically significant when *P* < 0.05.

## 3. Results

**Figure 3:**
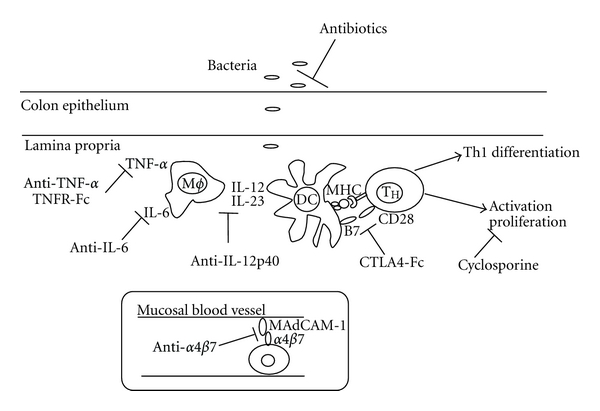
Inhibition of disease pathways in the adoptive transfer model. Schematic representation of drug targets.

In the following, the disease modifying effect of each of the investigated compounds in the adoptive transfer colitis model is presented. A schematic representation of the drug targets is shown in Figure 3. First, experimental data with the biologicals (i.e., monoclonal antibodies (mAb) and receptor fusion proteins (R-Fc)) are presented, followed by data from a number of compounds currently used to treat CD or UC. Although broad-spectrum antibiotics and metronidazole are mainly used for subgroups of IBD patients or for complications like pouchitis, we have included this treatment regimen, since the influence of the microflora in the pathogenesis of IBD is a central topic. Due to the large data material, readers are referred to the supplementary material for a complete presentation of data (Figure SF1 and Tables ST1–ST10 available at doi: 10.1155/2012/412178).

### 3.1. Rat Anti-Mouse TNF-*α* mAb Treatment

**Figure 4:**
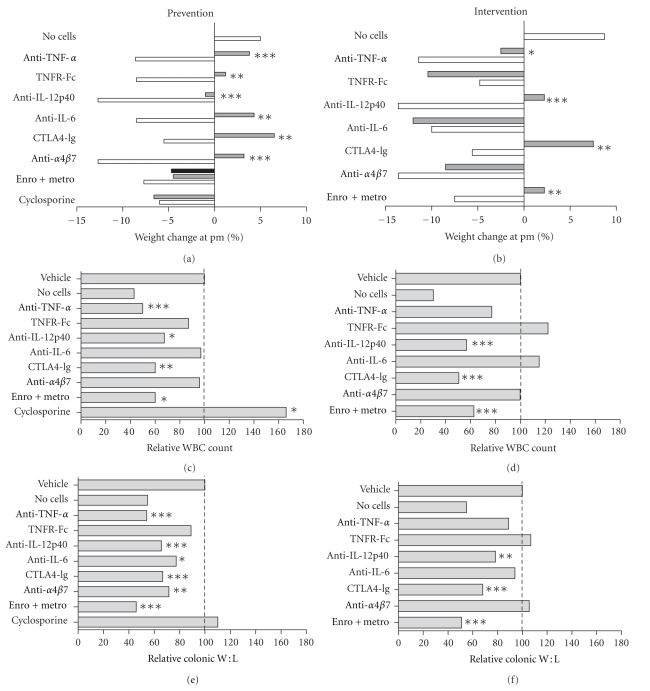
Changes in key disease parameters following treatment. Key disease parameters (weight loss, WBC counts, and colon W : L ratio) are depicted for preventive treatment (a–c) and interventive treatment (d–f). Disease parameters are shown as ((post mortem weight−start weight)/Start weight)∗100, (WBC count of drug/WBC count of control)∗100, (W : L ratio of drug/W : L ratio of control)∗100. (a and d) White bars represent vehicle control groups, grey bars represent treatment groups, and black bars represent mice which were not reconstituted but received treatment. (b, c, e, and f) Grey bars represent relative WBC counts and W : L ratios.

In the 28 day prevention study, mice treated with the isotype control began to loose weight after two weeks, while the weight curve for the rat anti-mouse TNF-*α* mAb-treated mice was comparable to that of healthy controls. At the end of the study, the anti-TNF-*α*-treated group had lost significantly less weight than the control group (*P* < 0.001, Figure 4, Tables 2 and ST1). Two mice in the control group were sacrificed due to extensive weight loss before the end of the study. The fecal score was increased in both groups but was significantly lower in the anti-TNF-*α* group (*P* < 0.001, Tables 2 and ST2). The WBC count in the anti-TNF-*α* group was almost as low as in the unreconstituted controls, while it was significantly higher in the isotype control group (*P* = 0.001, Table 2 and ST3). Similarly, the colonic W : L ratio (*P* < 0.001) and histological score (*P* < 0.0001) were significantly lower in the anti-TNF-*α* group compared to the isotype controls (Tables 2, SF1, ST4-5). In contrast to the prevention studies, intervention therapy with anti-TNF-*α* did not consistently ameliorate colitis in this model. Although the anti-TNF-*α*-treated group lost less weight than the control group (*P* < 0.05), none of the other clinical parameters were significantly affected by the treatment (Table 2 and ST6–10). Both for the fecal score, colonic W : L ratio and histological score (SF1), the group seemed equally divided into responders and non responders, that is, having high or low scores and values, respectively, rather than being equally distributed around the mean or median.

### 3.2. TNF-*α* Receptor Fc Treatment

We first tested the human TNFR-Fc fusion protein etanercept at a dose of 5 mg/kg in a 21 days prevention study and found no significant effect of the compound on any of the parameters analyzed (data not shown). In the subsequent 28 days prevention study with a dose of 50 mg/kg, this group had significantly less weight loss than the vehicle control group (*P* < 0.01), while the fecal score was slightly but not significantly lower (Figure 4, Tables 2 and ST1-2). One mouse in the vehicle control group was sacrificed before week four, due to extensive weight loss. There was no difference in the mean WBC count or colonic W : L ratio between the two groups. Since we found no effects of the compound (except from decreased weight loss) and since the colonic W : L ratio is highly predictable for the histological score (as described in [15]), no histological scoring was made. In the intervention study, TNFR-Fc (5 mg/kg) did not affect any of the measured parameters (Table 2 and ST6–10). Since the prevention study using 50 mg/kg did not have any effect either, this dose was not tested in intervention studies.

### 3.3. Rat Anti-Mouse IL-12p40 mAb Treatment

In the 28-day prevention study, mice treated with the isotype control began to loose weight after two weeks, while the weight curve for the rat anti-mouse IL-12p40 mAb-treated mice was comparable to that of healthy controls. At the termination of the study, mice treated with anti-IL-12p40 mAb had lost significantly less weight than the isotype control group (*P* < 0.001) (Table 2 and ST1). Similarly, anti-IL-12p40 mAb treatment resulted in significantly lower WBC count (*P* < 0.05), colonic W : L ratio and histological score (SF1) (*P* < 0.001 for both parameters), while it did not significantly improve fecal score (Tables 2 and ST2-S5). Intervention with anti-IL-12p40 mAb from day 21 reversed the progressive weight loss. At the end of the study, this group had lost significantly less weight than the control group (*P* < 0.0001) (Table 2 and ST6). The fecal score tended to be lower in the anti-IL-12p40 mAb group, and the colonic W : L ratio and histological score were significantly reduced compared to the isotype control (*P* < 0.01 and *P* < 0.05, resp., Tables 2, SF1, and ST7-10).

### 3.4. Rat Anti-Mouse IL-6 mAb Treatment

Preventive treatment with a rat anti-mouse IL-6 mAb reduced weight loss (*P* < 0.01) and fecal score (*P* < 0.01) but failed to significantly reduce WBC counts compared to the isotype control group (Table 2 and ST1–3). The colonic W : L ratio of the anti-IL-6 mAb treated group was significantly lower than that of the isotype treated group and was comparable to the unreconstituted healthy control group (Table 2 and ST4). Similarly, the histological score was significantly less in the anti-IL-6 mAb-treated group compared to the isotype control (*P* < 0.01, Tables 2, SF1, and ST5). Intervention with anti-IL-6 mAb from day 21 did not significantly affect any of the measured parameters (Tables 2, SF1, and S6-10).

### 3.5. Human CTLA4-Ig Treatment

Preventive treatment with human CTLA4-Ig effectively inhibited development of colitis. The weight curves for CTLA4-Ig-treated mice were comparable to the weight curves for the unreconstituted healthy control mice. The fecal score at the termination of the study (*P* < 0.01), the WBC count (*P* < 0.01), and colonic W : L ratio (*P* < 0.001) were significantly lower in the CTLA4-Ig groups compared to the isotype control group (Table 2 and ST1–4). Remarkably, no signs of inflammation were found in any of the animals, suggesting a very potent effect of the compound (Tables 2, SF1, and ST5).

A profound effect of CTLA4-Ig treatment on all the measured parameters was likewise identified in the intervention study. Shortly after initiation of treatment, the weight loss was reversed and the animals gained weight comparable to the unreconstituted. Likewise, the fecal score (*P* < 0.01), WBC count (*P* < 0.001), colonic W : L ratio (*P* < 0.001), and histological score (*P* < 0.001) were significantly lower than for the isotype control group at the end of the study (Tables 2, SF1, and ST6-10).

### 3.6. Rat Anti-Mouse *α*4*β*7 Integrin mAb Treatment

Preventive treatment with a rat anti-mouse *α*4*β*7 mAb diminished weight loss (*P* < 0.001) and fecal score (*P* < 0.05, Table 2). However, the number of WBC was not significantly changed (Tables 2 and ST2-3). Inhibition of T-cell homing to the gut by anti-*α*4*β*7 mAb treatment reduced colonic disease as indicated by the significantly lower colonic W : L ratio and histological score compared to the isotype control group (*P* < 0.01 for both parameters, Tables 2, SF1, and ST4-5). Intervention with anti-*α*4*β*7 mAb at day 21 did not significantly affect any of the measured parameters (Figure 4, Tables 2, SF1, and ST6–10).

### 3.7. Antibiotic (Enrofloxacin and Metronidazole) Treatment

Mice treated with antibiotics (unreconstituted and reconstituted) did not develop weight loss in a preventive setting. In contrast, reconstituted vehicle treated mice progressively lost weight (Figure 4). The fecal score was slightly increased by the treatment itself but was significantly lower in the reconstituted mice treated with antibiotics compared to the reconstituted vehicle group (*P* < 0.001) as was the WBC count (*P* < 0.05), the colonic W : L ratio (*P* < 0.001), and the histological score (*P* < 0.001, Tables 2, SF1, and ST2-5).

Intervention with antibiotics immediately reversed weight loss (Figure 4). At the termination of the study, the mice treated with antibiotics had lost significantly less weight than the vehicle control group (*P* < 0.01). Likewise, fecal score (*P* < 0.0001), WBC count (*P* < 0.001), colonic W : L ratio (*P* < 0.0001), and histological score (*P* < 0.001) were significantly lower than for the vehicle control group (Figure 4, Tables 2, SF1, and S7–10).

### 3.8. Cyclosporine Treatment

Cyclosporine had no effect on the degree of weight loss, fecal score, colonic W : L ratio or histological score, while the WBC count at necropsy was actually significantly higher in the cyclosporine group compared to the vehicle group (Table 2, SF1, and ST1-5). Since there were no effects of cyclosporine in the prevention study, the compound was not tested in intervention studies.

### 3.9. Summary of Experimental Data

Collectively, we found that CTLA4-Ig, anti-IL-12p40 mAb, and antibiotics prevented onset of colitis and cured established disease, while anti-TNF-*α* mAb, anti-IL-6 mAb, and anti-*α*4*β*7 mAb prevented onset of colitis but did not reverse established disease. Neither TNFR-Fc nor cyclosporine had any preventive or therapeutic effect in the current setup.

## 4. Discussion

The search for improved treatment opportunities against IBD is heavily dependent upon good animal models, both for efficacy studies and for understanding the underlying cause of the disease. To have any predictive value, the models must share central drivers of disease with the human disease they are representing. Intervention with some of the known main mechanisms in autoimmune disease (i.e., costimulation, T-cell homing, effect of cytokines, etc.) can teach us more about which pathways are central for the model. This allows one to select a model appropriate for the inflammatory pathway, the test compound is supposed to act on. The aim of our study was to estimate the preventive and therapeutic effect of a number of established or potential IBD drugs, using the SCID adoptive transfer colitis model. By using multiple drugs inhibiting potential or known pathogenic drivers in IBD, we sought after an increased understanding of the central drivers, in this specific model. Several of these compounds or their surrogate antibodies have previously been tested in colitis models, but never in the same study with essentially similar experimental setup and with compounds which are all commercially available. This allows a more precise comparison of the different compounds and thereby a better assessment of the model's predictive value. We chose the adoptive transfer colitis model not only because of its similarities with IBD (mainly CD), but also because the model has several practical advantages compared to other chronic colitis models in relation to pharmacological testing (e.g., the synchronized onset of disease, no generation of anti-drug antibodies and commercial availability of mice).

TNF-*α* is a key proinflammatory cytokine in IBD. The cytokine exerts its effects via activation of NF*κ*B and MAPK pathways, and subsequently induction of IL-6 and IL-1b, inhibition of T-cell apoptosis, chemoattraction, and so forth [25]. We found a significant preventive treatment effect of anti-TNF-*α* mAb, as has been previously described in the CD45RB^High^ model [11]. Our model setup suggests that TNF-*α* is most important in the beginning of disease since anti-TNF-*α* was largely effective in the prevention study. It is possible that the redundancy of the inflammatory cascades makes TNF-*α* less important when the inflammation is already established and CD4^+^ T cells have differentiated into a pathogenic effector phenotype. However, the human anti-TNF-*α* mAb's (adalimumab and infliximab) can induce remission in the majority of CD patients. These mAbs' have been reported to neutralize TNF-*α* as well as to induce apoptosis in T cells [26]. Whether the surrogate rat anti-mouse TNF-*α* mAb also has this dual function is unknown. As opposed to anti-TNF-*α* mAb treatment, there was no effect of TNFR-Fc in our studies, which is in accordance to the findings in CD [27].

IL-12p40 is predominantly produced by dendritic cells and phagocytes in response to microbial stimulation. It has a critical role in promoting the differentiation of naïve CD4^+^ T cells into mature T-helper effector cells. It is currently believed that IL-12 (p23/p40) and IL-23 (p19/p40) are central for the Th1 and the Th17 pathways, respectively, and both cytokines are inhibited by the anti-IL-12p40 mAb [16, 28]. We found that the anti-IL-12p40 mAb treatment was effective in our prevention as well as intervention setup, suggesting the importance of these pathways in the transfer model. Our observation is in agreement with previous results [20, 29, 30]. Significant clinical responses following treatment with an anti-IL-12p40 mAb have also been reported in CD patients [5, 31], and the drug is currently recruiting for a phase III trial in CD. Thus, our results suggest that the SCID adoptive transfer model is suitable for studying compounds targeting the IL-12p40 pathways.

IL-6 is a pleiotropic cytokine with a central role in immune regulation. Increased serum concentrations of IL-6 were reported to correlate with clinical activity of CD, and antibodies targeting IL-6 or IL-6R were efficacious in several animal models [16, 32, 33]. In accord, a humanized mAb against IL-6R showed promising results in a phase II study in active CD [6]. We found a significant therapeutic effect of anti-IL-6 in a preventive setting but not in the intervention study. As for the anti-TNF-*α* mAb treatment, this suggests that IL-6 is most important in the beginning, while the redundancy of the inflammatory cascades may make IL-6 less important when the inflammation is already established.

CTLA4-Ig binds to CD80/86 thereby preventing costimulation of T cells via CD28 [34]. CTLA4-Ig has shown therapeutic efficacy in several autoimmune diseases including rheumatoid arthritis, and in experimental models of autoimmune diseases [34, 35]. We found that CTLA4-Ig completely prevented colitis and very efficiently cured established disease, indicating a central role of T-cell costimulation in the model. However, shortly after completion of our experimental studies, clinical trials with CTLA4-Ig in UC and CD were terminated due to lack of efficacy [36]. The lack of negative regulation through CTLA-4 on, for example, Tregs may yield a therapeutic window which is not present in humans. Thus, the lack of important self-regulatory mechanisms must be taken into account when evaluating drugs targeting this specific pathway.

Neutralization of the integrin *α*4*β*7 on lymphocytes and monocytes inhibits homing of the cells to the gut via mucosal addressin cell adhesion molecule-1 (MAdCAM-1) on endothelial cells. We found that this could prevent the onset of disease in the transfer model as demonstrated previously [37]. However, once the colitis was already established, recruitment of leucocytes to the colon via this mechanism seemed no longer essential for maintenance of disease in our experimental setup. In contrast, resolution of established disease has been demonstrated in the cotton-top tamarin (spontaneous colitis) [38].

In phase II studies, Vedolizumab (targeting human *α*4*β*7) failed to meet the primary endpoint in CD patients with active disease, while there was a significant therapeutic effect in patients with UC [39, 40]. Natalizumab, which neutralizes *α*4 in conjunction with *β*7 as well as with *β*1, was effective for CD and is approved by the FDA to use in patients refractory to treatment with anti-TNF-*α* [41].

Thus, in IBD as well as in the models, there is not a clear-cut effect of inhibiting leukocyte homing via anti-*α*4*β*7, although it seems more effective in man than in the SCID transfer model. This could be due to the slower progression of the human disease, that is, there is a larger “window open for treatment” where recruitment of cells to the gut is still important. An alternative explanation is the involvement of *α*4*β*7 in homing to the small bowel and Payer's patches in Crohn's disease compared to *α*4*β*7 involvement in colonic CD4^+^ T cell infiltration in the SCID adoptive transfer model.

Although anti-*α*4*β*7 could not reverse established colitis in the model, the marked effect of the antibody in prevention studies suggests that the model is useful in studies of leukocyte homing to the colon via integrins and adhesion molecules.

Cyclosporine inhibits calcineurin, thereby inhibiting T-cell activation and proliferation. The compound is effective in patients with severe steroid-refractory UC [7, 42], while no controlled studies have shown effect of cyclosporine in CD [43]. We did not find any effect of preventive treatment with cyclosporine in accord with previous studies in transfer models [35, 44]. In contrast, cyclosporine significantly ameliorated acute DSS-induced colitis [19, 45]. Considering that the DSS model is mainly UC-like and the transfer model mainly CD-like (at least in terms of Th1/TH17 immunopathogenesis), data from experimental models are translational to the human disease. It is not clear why cyclosporine lacks effect in CD and in our model, but it has previously been shown that T cells are able to proliferate and exert effector functions via cyclosporine-resistant mechanisms [46–48]. In addition to its effect on T cells, cyclosporine has been shown to inhibit the effect of several other immune cells and proinflammatory mechanisms, which may account for its therapeutic effects in the acute DSS model, where T cells are not required for development of disease [49].

The normal intestinal microbial flora (microbiota) contributes significantly to the etiopathogenesis of IBD [3, 50], and antibiotics have in some studies been shown to induce and maintain remission in IBD patients [50]. However, the side-effects and the risk of developing microbial resistance associated with long-term treatment with antibiotics prevent the use of this treatment strategy. Instead, there is focus on developing microbial cultures, which can help bringing back the balance between the microbiota and the immune system [50]. We found that the combination of enrofloxacin and metronidazole completely prevented onset of colitis and cured established colitis in the adoptive transfer model, emphasizing the significance of the microbiota in this model. Thus, the experimental model and the human disease share this central factor in the immunopathogenesis, suggesting that the model may be useful in studies of the microflora's impact on disease. Combined treatment with metronidazole, which kills anaerobes and influences cell trafficking [51], was essential since enrofloxacin alone did not affect the disease (data not shown).

## 5. Concluding Remarks

We have evaluated the therapeutic effect of a number of IBD drugs and drug candidates in the SCID adoptive transfer colitis model and compared this to the therapeutic effect in IBD patients, when this information was available. The study shows that certain drivers of inflammation are shared between the model and the human diseases, that is, the cytokines IL-12p40, TNF-*α* and IL-6, the homing molecule *α*4*β*7, and the microbial flora. With regards to these drivers, the model seems to have a good predictive value.

However, our studies indeed also show limitations of the adoptive transfer model in this respect, since not all drugs effective against IBD are effective in the model. Following adoptive transfer, the development of disease is driven by the extreme expansion of the CD4^+^ cells in a lymphopenic host, and neither B cells nor CD8^+^ cells are present. Thus, some treatment effects in the preventive studies might be due to interfering with homeostatic expansion, which is not relevant in IBD and conversely, a test compound's effect on B and CD8^+^ T cells will have no effect in the model, although it might be effective in IBD. Moreover, compared to clinical trials where efficacy is evaluated, for example, 4–18 weeks post-initiation of treatment for some test compounds, an intervention period of two weeks may be too short to obtain a therapeutic effect in the model. This could be addressed with a model where the disease is progressing more slowly. This stresses the need of critically choosing for which types of experiments and compounds to use animal models and which of the models to use. It should be noted that there is a great variability in the disease pattern in IBD patients, and that patients respond differently to medication. The various experimental colitis models may represent different types and stages of severity of UC or CD [13] and altogether this makes the translation of data from experimental models to humans even more challenging.

## Supplementary Material

Supplementary figure 1 (SF1) depict the histologic appearence of the colon after treatment with componds or controls in preventive and interventive protocols. Supplementary table 1-10 (ST1-10) depict relative weight loss, fecal score, white blood cell counts, colonic weight length ratio and histological score after treatment with componds or controls in preventive and interventive protocols.

## Figures and Tables

**Table 1:** Study design—administration of compounds.

Compound	Dose (mg/kg)	cIg/Vehicle	Mice per group^1^	Dose/wk	Route
Rat anti-mouse TNF-*α*	25	rat IgG1	15^P^/10^I,2^	2	i.p.
Human TNFR-Fc (IgG1)	5–50	NaCl	10^P+I^	3	i.p.
Rat anti-mouse IL-12p40	25	rat IgG2a	10^P+I^	3	i.p.
Rat anti-mouse-IL-6	25	rat IgG1	10^p+I^	3	i.p.
Human CTLA4-Ig (IgG1)	10	hIgG1-Fc^3^	10^P+I^	3	i.p.
Rat anti-mouse-*α*4*β*7	25	rat IgG2a	10^P+I^	3	i.p.
Enro/metro^4^	350/875	H_2_O	9^P^/10^I^	daily	p.o.
Cyclosporine	25	H_2_O	10^p^	daily	p.o.

^1^Five to ten unreconstituted mice were included in addition to the compound and the control group.

^2^P: prevention, I: intervention.

^3^human IgG1-Fc.

^4^Treatment with enrofloxacin and metronidazole in the drinking water was initiated one week prior to transfer to let the mice adjust to the taste. Although we in pilot studies had identified a useful sugar mixture to mask the taste of metronidazole, the mice refused to drink and lost weight prior to adoptive transfer in the prevention study. Metronidazole was subsequently given orally by gavage once daily (this method was then also used for the intervention study).

**Table 2:** Statistics for all compounds. Clear lines represent prevention studies and bold represent intervention studies.

Compound	Weight change	Fecal score	WBC count	Colonic weight:length	Histological score
Anti-TNF*α*	<0.001	<0.001	<0.001	<0.001	<0.0001
	**<0.05**	**ns**	**ns**	**ns**	**ns**
TNFR-Fc^†^	<0.01	ns	ns	ns	—
	**ns**	**ns**	**ns**	**ns**	**—**
Anti-IL-12p40	<0.001	ns	<0.05	<0.001	<0.001
	**<0.0001**	**ns**	**0.001**	**<0.01**	**<0.05**
Anti-IL-6	<0.01	<0.01	ns	<0.05	<0.05
	**ns**	**ns**	**ns**	**ns**	**ns**
CTLA4-Ig	<0.01	<0.01	<0.01	<0.001	<0.05*
	**<0.01**	**<0.01**	**<0.001**	**<0.001**	**<0.001**
Anti-*α*4*β*7	<0.0001	<0.01	ns	<0.01	<0.01
	**ns**	**ns**	**ns**	**ns**	**ns**
Enro + Metro	ns	<0.001	<0.05	<0.001	<0.001
	**<0.01**	**<0.001**	**<0.001**	**<0.0001**	**<0.001**
Cyclosporine	ns	ns	<0.05**	ns	ns
	—	—	—	—	—

^†^Prevention = 50 mg/kg, intervention = 5 mg/kg.

*Used Wilcoxon signed rank test and compared with a hypothetical value of 0.0 since all scores were 0 in the CTLA4-Ig group.

**WBC count higher in treatment group—not analyzed.

## Abbreviations

CD: Crohn's disease
CTLA-4: Cytotoxic T-Lymphocyte Antigen 4
GM-CSF: Granulocyte macrophage colony-stimulating factor
Ig: Immunoglobulin
IBD: Inflammatory bowel disease
IL: Interleukin
IFN-*γ*: Interferon-gamma
cIg: Isotype controls
ns: Not significant
TNF-R: Tumor necrosis factor receptor
TNF: Tumor necrosis factor
UC: Ulcerative colitis
W : L: Weight-to-length ratio
WBC: White blood cell.
