# Supplementary material for: Pharmacological Evaluation of the SCID T Cell Transfer Model of Colitis: As a Model of Crohn's Disease
Source: Int J Inflam. 2012 Feb 19;2012:412178. doi: 10.1155/2012/412178 (PMC3328770; doi:10.1155/2012/412178)
Supplement: Supplementary file 1 — Supplementary figure 1 (SF1) depict the histologic appearence of the colon after treatment with componds or controls in preventive and interventive protocols. Supplementary table 1-10 (ST1-10) depict relative weight loss, fecal score, white blood cell counts, colonic weight length ratio and histological score after treatment with componds or controls in preventive and interventive protocols. [file 412178.f1.pdf]

Supplementary Table 1 (ST1)

Percent weight loss at post mortem (mean, SEM). Prevention study

Treatment	Cells + vehicle	Cells + Trx	Healthy	P value
Anti-TNF-α	-8.6 (2.5)	3.8 (0.9)	6.0 (1.5)	< 0.001
TNFR-Fc	-8.5 (2.2)	1.2 (2.0)	10 (0.6)	< 0.01
Anti-IL-12p40	-14(2.4)	-1.0 (0.9)	2.2 (1.0)	< 0.001
Anti-IL-6	-8.5 (2.5)	4.3 (2.5)	5.1 (2.6)	< 0.01
CTLA4-Ig	-5.5 (2.6)	6.5 (1.6)	6.4 (1.9)	< 0.01
Anti-α4β7	-14 (2.4)	3.2 (1.1)	2.2 (1.0)	< 0.0001
Enro + Metro	-7.7 (1.8)	-4.5 (1.0)	4.8 (1.8)*	ns
Cyclosporine	-6.0 (2.8)	-6.6 (2.8)	5.0 (1.6)	ns

*Unreconstituted, treated: -4.7 (1.7). Note that mice lost weight prior to transfer due to refusal to drink the antibiotics.

Supplementary Table 2 (ST2)

Fecal score at post mortem (median, min-max). Prevention study

Treatment	Cells + vehicle	Cells + Trx	Healthy	P value
Anti-TNF-α	3 (2-3)	2 (1-2)	0.5	< 0.001
TNFR-Fc*	3 (0-4)	1 (1-3)	0	ns
Anti-IL-12p40	2 (0-2)	2 (1-2)	0.5	ns
Anti-IL-6	2 (1-4)	1 (0-2)	1	< 0.01
CTLA4-Ig	2 (0-3)	1 (0-1)	0.5	< 0.01
Anti-α4β7	2 (0-2)	1 (0-1)	0.5	< 0.01
Enro + Metro**	2.5 (1-4)	1 (1-1)	0	< 0.001
Cyclosporine	2 (0-3)	2 (1-3)	0	ns

*Scores for week three. No improvement for TNFR-Fc group in week four.** Unreconstituted, treated: 1 (0-1)

Supplementary Table 3 (ST3)

WBC count x 10^9/L at post mortem (mean, SEM). Prevention study

Treatment	Cells + vehicle	Cells + Trx	Healthy	P value
Anti-TNF-α	2.7 (0.3)	1.3 (0.1)	0.8 (0.1)	< 0.001
TNFR-Fc	4.1 (0.4)	3.5 (0.5)	1.3 (0.2)	ns
Anti-IL-12p40	3.9 (0.4)	2.6 (0.2)	1.7 (0.4)	< 0.05
Anti-IL-6	3.3 (0.4)	3.2 (0.3)	1.7 (0.6)	ns
CTLA4-Ig	4.5 (0.4)	2.7 (0.2)	2.0 (0.2)	< 0.01
Anti-α4β7	3.9 (0.4)	3.7 (0.2)	1.7 (0.4)	ns
Enro + Metro*	3.6 (0.6)	2.2 (0.2)	2.2 (0.3)	< 0.05
Cyclosporine	3.2 (0.5)	5.3 (0.7)	1.5 (0.1)	< 0.05

* Unreconstituted, treated: 2.1 (0.5)

Supplementary Table 4 (ST4)

Colonic weight:length ratio (mean, SEM). Prevention study

Treatment	Cells + vehicle	Cells + Trx	Healthy	P value
Anti-TNF-α	53 (1.9)	29 (1.2)	28 (1.8)	< 0.001
TNFR-Fc	43 (2.3)	38 (2.3)	22 (1.0)	ns
Anti-IL-12p40	43 (3.0)	28 (1.7)	25 (1.5)	< 0.001
Anti-IL-6	44 (3.4)	34 (2.4)	23 (2.1)	< 0.05
CTLA4-Ig	45 (2.3)	30 (1.9)	23 (1.7)	< 0.001
Anti-α4β7	43 (3.0)	30 (1.2)	25 (1.5)	< 0.01
Enro + Metro*	40 (2.5)	18 (0.6)	24 (0.5)	< 0.0001
Cyclosporine	36 (3.1)	40 (1.7)	25 (1.4)	ns

* Unreconstituted, treated: 19 (1.2)

Supplementary Table 5 (ST5)
Histological score (median, min-max). Prevention study

Treatment	Cells + vehicle	Cells + Trx	Healthy	P value
Anti-TNF-α	9 (6-12)	1.5 (0-6)	0	< 0.0001
TNFR-Fc	ND	ND	ND	
Anti-IL-12p40	9.5 (6-12)	3 (0-6)	0	< 0.001
Anti-IL-6	6 (0-9)	0 (0-8)	0	< 0.05
CTLA4-Ig**	9 (0-11)	0 (0-0)	0	< 0.05
Anti-α4β7	9.5 (6-12)	4 (3-8)	0	< 0.01
Enro + Metro*	9 (5-9)	0 (0-3)	0	< 0.001
Cyclosporine	9 (4-12)	9 (7-10)	0	ns

ND: Not determined. *Unreconstituted, treated: 0 (0-3).**Used Wilcoxon Signed Rank test and compared with a hypothetical value of 0.0 since all scores were 0 in the CTLA4-Ig group.

Supplementary Table 6 (ST6)
Percent weight loss at post mortem (mean, SEM). Intervention study

Treatment	Cells + vehicle	Cells + Trx	Healthy	P value
Anti-TNF-α	-11 (2.9)	-2.5 (2.5)	9.2 (1.3)	< 0.05
TNFR-Fc	-4.8 (3.3)	-10 (2.9)	8.0 (2.4)	ns
Anti-IL-12p40	-14 (2.1)	2.2 (1.7)	9.2 (1.3)	< 0.0001
Anti-IL-6	-10 (3.2)	-12 (2.8)	5.1 (2.6)	ns
CTLA4-Ig	-5.6 (3.4)	7.5 (1.4)	9.2 (1.3)	< 0.01
Anti-α4β7	-14 (2.1)	-8.5 (2.9)	9.2 (1.3)	ns
Enro + metro	-7.5 (1.9)	2.2 (1.8)	9.4 (2.2)	< 0.01

Supplementary Table 7 (ST7)
Fecal score at post mortem (median, min-max). Intervention study

Treatment	Cells + vehicle	Cells + Trx	Healthy	P value
Anti-TNF-α	2 (1-4)	2 (0-3)	1	ns
TNFR-Fc	4 (2-4)	3 (0-3)	0	ns
Anti-IL-12p40	2 (1-3)	1.5 (0-2)	1	ns
Anti-IL-6	2 (1-3)	2 (1-3)	1	ns
CTLA4-Ig	2 (0-3)	0.5 (0-1)	1	< 0.01
Anti-α4β7	2 (1-3)	2 (1-3)	1	ns
Enro + metro	3 (2-4)	1 (0-1)	1	< 0.0001

Supplementary Table 8 (ST8)
WBC count x 10^9/L at post mortem (mean, SEM). Intervention study

Treatment	Cells + vehicle	Cells + Trx	Healthy	P value
Anti-TNF-α	5.4 (0.5)	4.2 (0.6)	1.3 (0.2)	ns
TNFR-Fc	4.6 (0.8)	5.7 (0.6)	1.6 (0.3)	ns
Anti-IL-12p40	4.9 (0.5)	2.8 (0.3)	1.3 (0.2)	0.001
Anti-IL-6	3.4 (0.4)	3.9 (0.4)	1.7 (0.6)	ns
CTLA4-Ig	4.8 (0.4)	2.4 (0.3)	1.3 (0.2)	< 0.001
Anti-α4β7	4.9 (0.5)	4.9 (0.6)	1.3 (0.2)	ns
Enro + metro	4.7 (0.3)	3.0 (0.3)	1.3 (0.5)	< 0.001

Supplementary Table 9 (ST9)
Colonic weight:length ratio (mean, SEM). Intervention study

Treatment	Cells + vehicle	Cells + Trx	Healthy	P value
Anti-TNF-α	45 (2.6)	40 (3.4)	21 (2.1)	ns
TNFR-Fc	43 (2.6)	46 (2.1)	28 (2.9)	ns
Anti-IL-12p40	41 (2.2)	32 (1.0)	21 (2.1)	< 0.01
Anti-IL-6	50 (3.1)	47 (2.9)	23 (2.1)	ns
CTLA4-Ig	43 (2.4)	30 (2.0)	21 (2.1)	< 0.001
Anti-α4β7	41 (2.2)	43 (1.8)	21 (2.1)	ns
Enro + metro	53 (3.4)	27 (1.6)	27 (1.9)	< 0.0001

Supplementary Table 10 (ST10)

Histological score (median, min-max). Intervention study

Treatment	Cells + vehicle	Cells + Trx	Healthy	P value
Anti-TNF-α	11 (4-13)	8.5 (0-12)	0 (0-0)	ns
TNFR-Fc	ND	ND	ND	
Anti-IL-12p40	11 (3-13)	4.5 (0-10)	0 (0-0)	< 0.05
Anti-IL-6	7 (5-10)	9 (5-12)	0 (0-0)	ns
CTLA4-Ig	10 (8-13)	4.5 (0-9)	0 (0-0)	< 0.001
Anti-α4β7	11 (3-13)	10 (7-13)	0 (0-0)	ns
Enro + metro	11 (3-12)	0 (0-6)	0 (0-0)	< 0.001

Prevention / Intervention

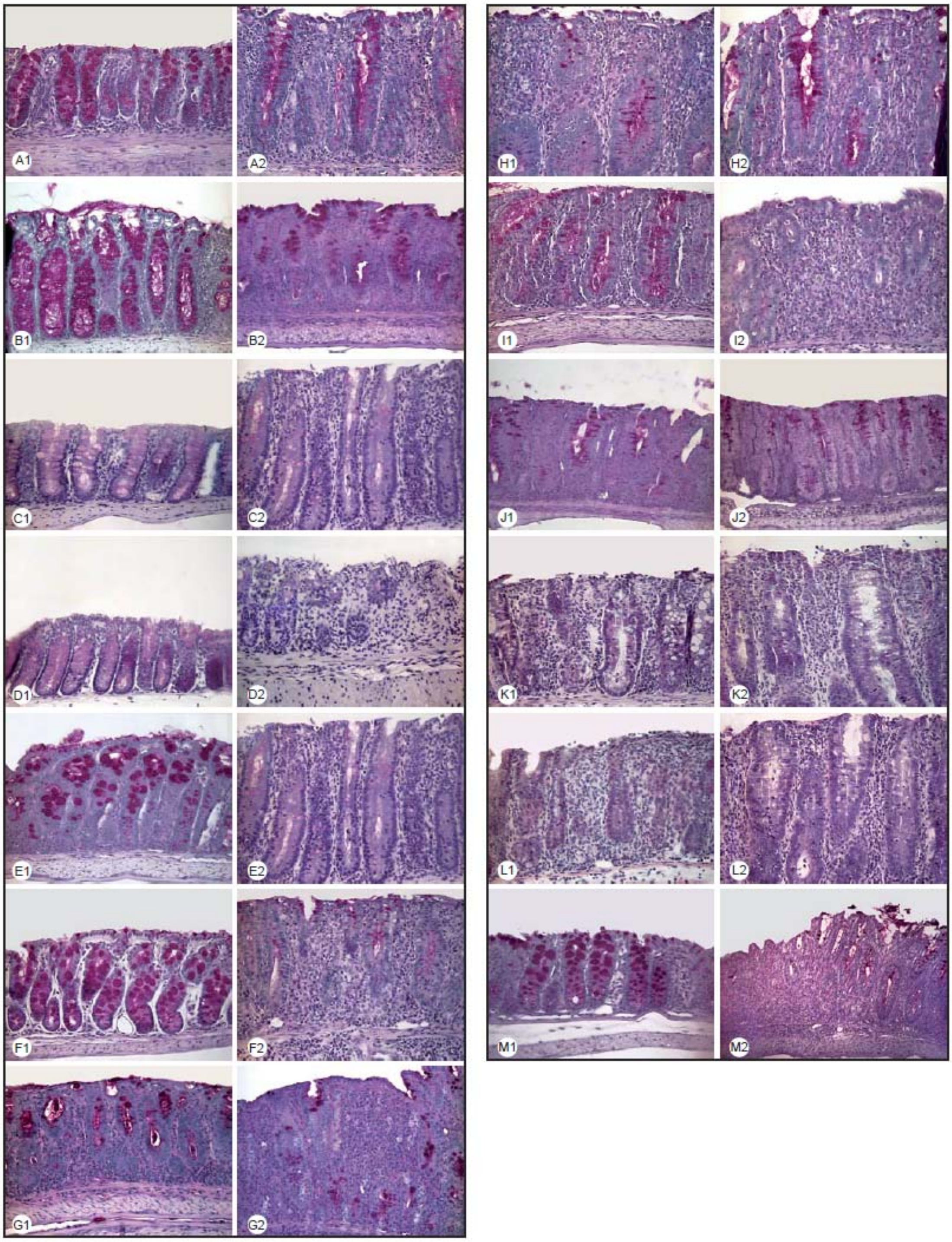

Supplementary figure 1 (SF1): Histopathologic appearance of colon after treatment with compounds or controls. Photomicrographs representative of the mean score of the individual groups. A1-G1 and A2-G2 are from the groups treated with compounds (1) and controls (2), respectively in the prevention study. H1-M1 and H2-M2 represent the compound (1) and control (2) groups in the intervention study. A, H: anti-TNF-α, B, I: anti-IL-12p40; C, J: anti-IL-6; D, K: CTLA4-Ig; E, L: anti-α4β7; F, M: enrofloxacine and metronidazole; G: cyclosporine. H&E PAS stain, original magnification x 25.
